# Possible role of artificial intelligence in diagnosis of cases with non-specific signs and symptoms of dengue: A comment

**DOI:** 10.1016/j.clinsp.2024.100388

**Published:** 2024-05-15

**Authors:** Marcos Roberto Tovani-Palone, Filippo Bistagnino, Jacopo Rosso Antonino, Arunkumar Subramanian

**Affiliations:** a Department of Pharmacy and Pharmacology, SRM College of Pharmacy, SRM Institute of Science and Technology, Kattankulathur, Chennai, India; b Department of Medical Biotechnology and Translational Medicine, International Medical School, Università degli Studi di Milano, Milan, Italy; c Department of Biomedical Sciences for Health, Università degli Studi di Milano, Milan, Italy; d Department of Pharmacology, SRM College of Pharmacy, SRM Institute of Science and Technology, Kattankulathur, Chennai, India

Given the global concern about the continued spread of dengue, which now also affects countries in the Northern region of the world, especially Europe,^1^^,^^2^ tools based on Artificial Intelligence (AI) have been developed with the aim of predicting possible scenarios and better directing related preventive actions.^3^^,^^4^ According to the European Centre for Disease Prevention and Control, dozens of locally transmitted cases of the infection were reported in Italy and France at the end of October 2023, while a smaller but still worrying number occurred in Spain in the same period. This has not only been restricted to the southernmost regions of Europe but has also affected the Auvergne-Rhône-Alpes and Île-de-France regions.^2^ Within this increasingly challenging context, there should be a persistent concern about how to improve different aspects of epidemiological and laboratory surveillance of the disease, mainly aiming for better diagnosis outcomes and appropriate treatment.

In this way, some critical issues should not be neglected, especially when taking into account the existence of different types of dengue, with variable symptoms, in addition to the occurrence of many cases whose clinical manifestations do not have specificity with the standard clinical diagnostic criteria.^5^ As a result, significant challenges persist for both clinical diagnosis and predictability of disease occurrence, thus requiring timely confirmation through laboratory tests. This in turn can have an important impact on the prevalence of underreporting and errors in diagnosis and management, which may involve a significant percentage of cases of the disease around the world. In this respect, the possible use of AI to predict diagnoses and assist in the decision to indicate confirmatory laboratory tests for cases with non-specific signs and symptoms amidst an outbreak or epidemic of the disease could play an important role in both early and differential diagnosis, and consequently in the epidemiological setting.

A further important point in this regard is that considering the potential role of AI in diagnosis, the main contribution of this powerful tool would be particularly related to the well-known difficulty in differentiating tropical infections due to their homogeneous nature, and clinical and laboratory presentations. Based on this premise, a recent and relevant related study has identified by retrospective analysis the best predictors in terms of laboratory parameters and clinical presentations of different tropical infections to develop a multinomial logistic regression model and a machine learning model to assist in differential diagnosis.^6^

Furthermore, the use of AI tools to indicate the need for different confirmatory laboratory tests in cases with non-specific clinical features, including the rapid test (NS1 antigen detection), IgM and IgG antibody tests or molecular test (Real-Time Polymerase Chain Reaction, RT-PCR), could help healthcare teams involved to make related decisions with greater levels of accuracy and assertiveness. In this connection, the time of infection, specific and non-specific signs and symptoms, current medical history and antecedents, epidemiology, and specific physical examinations should be essential factors to be considered. Such an approach would be a great advantage, especially during outbreaks/epidemics of the disease. In these situations, in addition to the overload of hospitals and outpatient services, delays in the release of relevant test results may also occur in response to excessive workload or insufficient laboratory infrastructure. Other possible related benefits are described in Table 1, summarizing the present propositions.

**Table 1:** Potential additional advantages of using AI to predict the need for confirmatory laboratory tests for cases with non-specific signs and symptoms of dengue.

1. Enable healthcare providers to detect the disease earlier
2. Provision of an improved surveillance of diseases from diverse sources
3. Reduce the cost involved in accompanying unnecessary diagnostic tests
4. Assist healthcare providers in designing new treatment strategies and/or protocols
5. Improve patient experience during outbreaks or epidemics
6. Identification of other potential specific clinical patterns of signs and symptoms useful for differential diagnosis
7. Improve patients’ risk stratification through a combination of patients’ medical background and viral species features

Source: Elaborated by the authors.

Looking toward advances in early diagnosis of the disease using AI, different prediction models have been studied in order to forecast the onset of dengue infection.^7^ Furthermore, there has also been a concern for the classification of microarray datasets**.** In this sense, the results of an important study found that AI-based methods for the selection of features, although showing differences in their performance, may be helpful in improving the classification accuracy of dengue data and even in decreasing the computational time required for the selection process, thus leading to a better understanding of the data.^8^ AI models may also play a pivotal role in understanding the dynamics of disease prognosis through the integration of analysis, including inpatient and outpatient management of dengue cases.^9^

Following this line of reasoning, another prominent application of AI techniques is related to the integration of epidemiological data with the purpose of assisting in targeted interventions for disease surveillance and control.^4^ A growing body of scientific evidence suggests that environmental factors such as rainfall, humidity, and temperature play a crucial role in the spreading of dengue. Integrating these factors using machine learning methods can improve the performance of predicting its incidence.^10^ Practical evidence of this approach is highlighted by Anggraini Ningrum et al. who obtained an AI-based model capable of predicting an outbreak with an accuracy of up to 89.25 %.^11^ Indeed, such methods can function as a supplementary tool for traditional surveillance, leading to new perspectives in advanced analysis within the field of public health.^12^ Thus, AI may potentially aid in the comprehensive understanding of the disease, leading to more effective dengue control strategies and management.

Finally, more research involving AI could therefore be useful for the development of tools aimed at improving both the diagnosis and management of dengue cases, while contributing to technological advances in healthcare and the well-being and quality of life of the population.

## CRediT authorship contribution statement

**Marcos Roberto Tovani-Palone:** Conceptualization, Writing – original draft, Writing – review & editing, Supervision. **Filippo Bistagnino:** Conceptualization, Writing – original draft, Writing – review & editing, Supervision. **Jacopo Rosso Antonino:** Writing – original draft. **Arunkumar Subramanian:** Writing – original draft.

## Conflicts of interest

The authors declare no conflicts of interest.
